# Report from the Scientific Poster Session at the 12th Annual Cardiometabolic Health Congress in Boston, USA, 4–7 October 2017

**DOI:** 10.3390/medsci5040037

**Published:** 2017-12-19

**Authors:** Organizing Committee CMHC 2017

**Affiliations:** Tarsus Cardio dba Cardiometabolic Health Congress (CMHC), Boca Raton, FL 33431, USA; info@cardiometabolichealth.org; Tel.: +888-997-0112

## 1. Preface

More than one third of the population has at least one cardiometabolic risk factor-dyslipidemia, cardiovascular disease (CVD), hypertension, diabetes, and/or obesity. In its 12th year, the Cardiometabolic Health Congress (CMHC) is the largest, US-based, multidisciplinary conference focused solely on the management of cardiometabolic risk and the prevention of cardiovascular and metabolic disease, and is chaired by top experts: Christie M. Ballantyne, MD; Robert H. Eckel, MD; George L. Bakris, MD; and Jay S. Skyler, MD. The three and a half-day event was attended by over 1000 cardiologists, endocrinologists, lipidologists, and allied healthcare professionals from across the world and offered a one-of-a-kind opportunity to learn real-world solutions to integrate immediately into clinical practice.

The 2017 Cardiometabolic Health Congress was successful in providing up-to-date and clinically relevant education to clinicians. Several innovative and informative sessions were offered during the congress, including Business of Medicine sessions focusing on the patient-centered medical home, the preauthorization process, patient adherence, and increasing practice revenue. The congress kicked off with the widely-popular Food and Drug Administration (FDA) Updates and Late Breaking Clinical Trials session where the attendees learned the latest developments in key cardiometabolic topics. Featured sessions highlighted an array of topics including triglyceride and high-density lipoprotein cholesterol (HDL-C) management, the involvement of micro and macronutrients in cardiometabolic disorders, the effect of the circadian rhythm on obesity and CVD, and new insights into insulin resistance. World-renowned speakers presented throughout the meeting, including keynote Stanley Hazen, MD; C. Ronald Kahn, MD; Barbara Kahn, MD; Paul M. Ridker, MD; Eugene Braunwald, MD; and Marc S. Sabatine, MD.

In addition to offering cutting-edge and comprehensive education, the 2017 CMHC hosted its first annual Scientific Poster Session, where investigators from around the world brought the latest data from current research and clinical findings to share with attendees.

## 2. Keynote Poster Abstracts

### 2.1. Evaluation of Endothelial Function in Patients with Type 2 Diabetes Mellitus Attending a Tertiary Care Medical Centre in South India

PingaliUsharaniNutalapatiChandrasekharKoilagundlaNiranjanDepartment of Clinical Pharmacology and Therapeutics, Nizam’s Institute of Medical Sciences (NIMS), Panjagutta, Hyderabad, Telangana 500082, India

**Purpose:** Diabetes mellitus (DM) is associated with increased risk of cardiovascular (CV) disease. In normal healthy endothelium, there is a balance between the release of vasodilatory and vasoconstrictive factors. Endothelial dysfunction (ED) is characterized by loss of endothelium dependent vasodilatation and can be considered as a major step in atherogenesis. Decreased production, increased degradation, or decreased sensitivity to nitric oxide are involved in ED. Early signs of diabetic vasculopathy include impaired endothelial function and increased arterial stiffness, which are independent predictors of CV events. Testing ED noninvasively using digital pulse photoplethysmography (PPG) helps in the evaluation of patients with CV risk. Thus, the present study was designed to evaluate the ED by endothelial dependent vascular response to salbutamol and measure the reflection index (RI) in type 2 diabetes mellitus patients at a tertiary care medical center. 

**Methods:** Drug-naive type 2 diabetic subjects and controls were enrolled for evaluation after written informed consent to ethics committee-approved protocol. Subjects’ demography and medical history were recorded. Laboratory tests for clinical chemistry, high-sensitivity C-reactive protein (hsCRP), lipids, and glycated hemoglobin (HbA1c) were conducted. Endothelial function determined by endothelial dependent vascular response to salbutamol challenge was assessed by change in RI using PPG, non-invasive method for deriving digital pulse volume (DPV) by measuring the transmission of infrared (IR) light through the pulp of the finger (index finger). After fasting overnight, subjects rested for about half an hour in a temperature- and humidity-controlled room. Vital parameters were recorded. Baseline diphosphoglycerate (DPG) and three measurements of RI (Reflection index = b/a × 100) were recorded; mean values were taken. Subjects then inhaled 400 mcg salbutamol through a spacer. Fifteen minutes later, three readings of RI were noted, and the mean was calculated. The percentage change in mean RI from baseline was recorded. Endothelial dysfunction was defined as RI < 6% (salbutamol challenge test). 

**Results:** In the present study, 152 diabetic subjects (82 males (M); 70 females (F)) and 148 (76 M; 72 F) matched controls were included. Body mass index (BMI) was significantly higher in diabetics (26.2 ± 3.4 kg/m^2^) than healthy controls (21.3 ± 2.9 kg/m^2^). Differences between biochemical laboratory parameters were statistically highly significant between subjects and controls. In the control group, the values were within normal range, but diabetic subjects had higher plasma glucose; HbA1c; total cholesterol; triglycerides; low-density lipoprotein (LDL); and lower HDL values. The hsCRP was also higher at 8.1 ± 1.4 mg/dL. Blood urea, serum creatinine, and hepatic parameters were within normal range. The mean change in reflection index (RI%) was considered as a marker of endothelial function and endothelial dependent vasodilator response. The change in RI was −14.2 ± 1.2% (pre-salbutamol 64.9 ± 8.6% to 50.7 ± 9.8% post-salbutamol), indicating normal response. In diabetic subjects, however, the response pre-salbutamol was 60.6 ± 9.6% and post-salbutamol was 58.2 ± 8.6%; with the mean RI being −2.4 ± 0.8%, indicating endothelial dysfunction. 

**Conclusions:** Advances in the assessment of endothelial function in the last decades, mainly through the non-invasive techniques, have facilitated either basic or clinical researches in cardiovascular and endocrine fields. The results of the present study highlight the importance of the reflection index in identifying vascular damage in subjects with diabetes mellitus who have high cardiovascular risk. The change in reflection index was found to be a good, sensitive, simple, and reliable method for assessing subjects with increased CV risk, which also serves as a non-invasive independent predictor of cardiovascular morbidity. Much evidence suggests that endothelial dysfunction is the main etiological factor for micro and macro vascular complications in DM. Hence, identifying and correcting endothelial dysfunction helps in reducing cardiovascular morbidity. Further long-term studies in diabetic subjects investigating the assessment of the reflection index for the evaluation of the reversal of endothelial function with therapeutic intervention are suggested.

### 2.2. A Study of Highly Standardized Aqueous Extract of Terminalia Chebula 250 mg, 500 mg versus Placebo in Modifying Cardiovascular Risk with Special Reference to Endothelial Dysfunction in Patients with Type 2 Diabetes Mellitus

PingaliUsharani.NutalapatiChandrasekharKoilagundlaNiranjanDepartment of Clinical Pharmacology and Therapeutics, Nizam’s Institute of Medical Sciences (NIMS), Panjagutta, Hyderabad, Telangana 500082, India

**Purpose:** Cardiovascular disease is a major cause of morbidity and mortality among patients with diabetes mellitus (DM), and these patients account for a significant proportion of all patients with cardiovascular disease. Other risk factors are hypertension, high low-density lipoprotein (LDL) cholesterol, low high-density lipoprotein (HDL) cholesterol, and oxidative stress. Endothelial dysfunction (ED) is one of the potential contributors to the pathogenesis of vascular disease in DM. Diabetes is associated with an increased risk of atherosclerosis, including platelet hyper-reactivity increased inflammation and endothelial dysfunction. Ayurvedic and many herbal drugs have been used for centuries in India. They possess potent antioxidant, anti-inflammatory, and cardioprotective properties and are used by patients with increased risk of cardiovascular morbidity and mortality. *Terminalia chebula* (TC) is reported to possess antidiabetic, antioxidant, and anti-inflammatory activity, amongst others. *Terminalia chebula* fruit extract shows antioxidant effect by strongly inhibiting the in vivo lipid peroxidation in a rat liver study. Lipid peroxides may be pro-inflammatory and can damage tissue directly. A previous study reported that the oral administration of *Terminalia chebula* extract once daily for two months reduced elevated blood glucose, and significantly reduced increases in glycosylated hemoglobin (HbA1c). However, very less information is available about the effects of TC in human diabetic patients. The present study was thus undertaken to evaluate the effect of TC 250 mg, TC 500 mg versus placebo on endothelial function in patients with type 2 diabetes.

**Methods:** Patients of either gender, aged 30–65 years, with a fasting plasma glucose of 110–126 mg/dL, HbA1c between 6.5% and 8%, and on stable dose of anti-diabetic medication (metformin 1500–2500 mg/day) for the past 8 weeks prior to the screening visit; and having endothelial dysfunction defined as ≤6% change in reflection index (RI) on the post-salbutamol challenge test were included in the study. Patients with severe uncontrolled hyperglycemia, uncontrolled hypertension, cardiac arrhythmia, impaired hepatic or renal function, or any other serious disease requiring active treatment and treatment with any other food supplements were excluded. 

All the eligible subjects were randomized to receive either treatment for 12 weeks—group 1 was given one capsule of *Terminalia chebula* 250 mg twice daily; group 2 was given one capsule of TC 500 mg twice daily; and group 3 was given one capsule of placebo twice daily. 

Subjects were reviewed for follow up at 4, 8, and 12 weeks of therapy. At each visit, they were evaluated for efficacy and safety. Pharmacodynamic evaluation for endothelial function was conducted at baseline and end of treatment. Salbutamol challenge test employing digital volume plethysmography was used to assess endothelial function. Blood samples were collected for biomarkers (nitric oxide, malondialdehyde (MDA), glutathione, high-sensitivity C-reactive protein (hsCRP)) and serum lipid profile, before and at end of treatment. Safety lab investigations for hematological, hepatic, and renal biochemical parameters were conducted before and at the end of the study. Any adverse drug reaction (ADR) reported was recorded in the case report form. Compliance was assessed by the pill count method. ANOVA, *t*-test and Tukey’s post hoc tests were used for statistical analysis by Prism Graphpad.

**Results:** Of the 74 subjects screened, 60 eligible subjects completed the study, 20 in each group. Significant reduction in mean RI was noted with TC 250 mg (−2.38 ± 0.82% to −4.93 ± 1.87%; *p* < 0.001) and TC 500 mg (−2.35 ± 0.85% to −6.14 ± 1.01%; *p* < 0.001), suggesting improvement in endothelial function compared to baseline and placebo (−2.11 ± 1.61% to −1.01 ± 2.05%). On further analysis, it was found that change in RI with TC 500 mg was significantly better compared to 250 mg (*p* < 0.001). 

Significant improvement was noted in biomarkers of oxidative stress and systemic inflammation with TC 250 and 500 mg compared to baseline and placebo. The mean percent change in biomarkers was analyzed for each treatment. It was observed that the mean increase in nitric oxide was 10.64 ± 6.16% for TC 250 mg and 16.52 ± 5.19% for TC 500 mg compared to baseline, while glutathione was 9.25% and 17.97%, respectively, for TC 250 mg and TC 500 mg compared to baseline. The mean percentage reduction in malondialdehyde was 6.83% with TC 250 mg and 11.20% with TC 500 mg. Similarly, TC 250 mg and TC 500 mg produced a mean percentage decrease in hsCRP of 9.25% and 26.47%, respectively, compared to baseline. There was no significant change in any of the parameters with placebo. 

Treatment with TC 250 mg and 500 mg significantly reduced total cholesterol (*p* < 0.001), LDL-cholesterol (LDL-C, *p* < 0.01), and triglycerides (*p* < 0.001) levels compared with baseline. There was an increase in HDL-cholesterol (HDL-C, *p* < 0.001) levels seen with TC 500 mg compared with baseline. 

All treatments were well tolerated with no serious adverse event. Two patients on TC 250 mg and one patient on TC 500 mg complained of dyspepsia. Mild headache was reported by three patients in placebo. All ADRs resolved with symptomatic treatment. None of the patients discontinued the study prematurely because of these adverse events.

**Conclusions:** Both TC250 and 500 mg significantly improved endothelial function and serum lipid profile as well as reduced biomarkers of oxidative stress, suggesting improvement in endothelial function in diabetic patients. No significant alterations were observed in safety lab parameters. *Terminalia chebula* 500 mg twice daily produced more pronounced response on pharmacodynamic parameters of endothelial function and biomarkers of oxidative stress as evidenced by a significant reduction in mean RI index and improvement in nitric oxide, glutathione, and hsCRP compared to TC 250 mg and Placebo. It is suggested that further studies maybe undertaken in a larger number of patients to continue to explore the beneficial effects of these formulations.

### 2.3. Self-Image, Obesity, and African-American Women

CollinsAlkieshaLeafmanJoanA.T. Still University, Kirksville, MO 63501, USA

**Purpose:** Obesity has become a worldwide epidemic, and the percentage of African-American women is among the highest diagnosed as overweight or obese. Obesity increases the risks of co-morbidities as well as negative psychological effects. There is a discrepancy between the medical community and minority populations, specifically African-Americans, as to what is physiologically considered obese or overweight. The purpose of this quantitative descriptive study is to examine the relationship between an obesity diagnosis and the self-image of college-educated African-American women.

**Methods:** The survey used for this study was modified using three body image surveys with the approval of the creator of the original surveys. The final survey included demographic, body image, and self-image variables. This survey captured self-perceptions of body and self-image in different environments, as well as how the judgment of others affected participants’ impressions of themselves.

This quantitative descriptive study was approved by A.T. Still University’s Institutional Review Board and the survey was uploaded to Survey Monkey.

African-American women were recruited from one of four predominately African-American sororities, identified as Sorority X. There were a total of 98 respondents, with 66 that matched the inclusion criteria. Demographics requested were self-reported indications of race, age, education level, height, weight, and body mass index (BMI).

Data was retrieved after 13.5 weeks and downloaded from Survey Monkey to SPSS on a password-protected personal computer owned by the primary investigator, and analysis was conducted using IBM SPSS Version 23. 

Descriptive statistics, including frequency, percentage, mean, standard deviation, and minimum and maximum were conducted for all variables, and a Wilk-Shapiro test was conducted to determine normality. Results revealed distribution to be uneven; therefore, non-parametric testing was performed. 

A two-tailed Spearman correlation test was conducted to examine the relationship between the output variable and the ordinal dependent variables. Alpha level was set at *p* = 0.05. Upon completion of the output variable analysis, additional correlations between independent variables were further explored and analyzed to examine significant relationships.

**Results:** Results showed that 53% (*n* = 35) of overall participants strongly and very strongly felt an obesity diagnosis affected their self-image, while 4.5% (*n* = 3) indicated that an obesity diagnosis did not affect their self-image at all. Thirteen (19.7%) participants reported an obesity diagnosis minimally affected their self-image. 

Eighty-seven percent (*n* = 57) of participants strongly agreed that they had thoughts of being thinner or reducing body fat, and 78.8% (*n* = 52) of participants agreed that they felt pressure by the media to look thinner and reduce their body fat. 

Of the participants, 40.9% (*n* = 27) did not compare their body sizes to others when they were eating at a restaurant, but the results showed a strong correlation to participants’ overall self-image when comparing their body size to others at a restaurant (0.361). 

Results showed that participants strongly agreed that most pressure to be thin and reduce body fat came from the participants themselves (87%) and the media (47%) as compared to pressure to be thin and reduce body fat from family (15.2%) and peers (9.1%).

Thirty (45.5%) participants reported that the opinions of others were never important to them regarding their body size, and 15 (22.7%) always thought, wondered, or were bothered by what other people thought of their body size.

Thirty-five (53.1%) participants reported they disagreed and strongly disagreed that their peers’ thoughts of them being thinner affected their self-image, and 36 (55.5%) reported they disagreed and strongly disagreed that their peer’s thoughts of reducing their body fact affected their self-image.

Twenty-eight (42.4%) participants reported that, when meeting new people, they never wondered what the person thought of their body size, and 30 (45.5%) participants reported that they never thought the opinion of others regarding their body size was important.

**Conclusions:** This research study revealed, for the population tested, that a medical diagnosis of obesity using only the BMI tool does affect the overall self-image of college-educated, African-American women, 30–45 years old, especially when participants compared their body size and idea of being thinner to strangers, friends/peers, family, and images in the media. The results of this study could be important for encouraging providers to consider the psychological component of an obesity diagnosis and how it affects the way African-American women view themselves. The potential benefit to society regarding this study would be an awareness of the effect of obesity on the self-image of African-American women. The discrepancy of the African-American female’s perception of body image as it relates to obesity is paramount to understanding how to address this topic in the African-American community and should be further studied.

### 2.4. Obstructive Sleep Apnea and Atrial Fibrillation: A Meta-Analysis

YoussefIriniKamranHaroonPatelNiravYacoubMenaGoulbourneCliveKumarShwetaJinaNimiraDesulmeBertnard BKaneJesseMcFarlaneSamy I.Department of Medicine, State University of New York Downstate Medical Center, Brooklyn, NY 11203, USA

**Purpose:** Atrial fibrillation (AF) is the most common sustained arrhythmia leading to cardiovascular morbidity and mortality. As the incidence of AF continues to rise, it is imperative to identify and treat potentially modifiable risk factors for the disease. While the traditional risk factors for AF include hypertension, diabetes, and coronary atherosclerosis, among others, obstructive sleep apnea (OSA) and sleep disordered breathing (SDB) are emerging disorders that appear to be important and rather treatable risks factor for AF. Proposed mechanisms for AF in OSA/SDB populations include increased intrathoracic pressures, which in turn lead to impaired left ventricular relaxation and atrial stretching. These mechanisms together with hypoxia and hypercapnia result in atrial fibrosis and tachyarrhythmia. Nevertheless, conflicting evidence exists in the literature regarding OSA/SDB as an underlying cause of AF. Therefore, we conducted a meta-analysis of all available studies to characterize the relationship between OSA-SDB and AF.

**Methods:** Databases including PubMed, Medline, and Cochrane Library were searched for relevant studies using the keywords “atrial fibrillation”, “obstructive sleep apnea”, and “sleep disordered breathing”. OSA was categorized by an apnea-hypopnea index (AHI) >5, a respiratory distress index (RDI) >30, or a 3% oxygen desaturation index (ODI) >15. AF was diagnosed by electrocardiogram (ECG) studies. All subjects included had an established diagnosis of OSA using the abovementioned criteria. Within these subjects, the occurrence of AF versus no AF was then compared. The pooled data was analyzed using Comprehensive Meta-Analysis package V3 (Biostat, USA). The Mantel-Haenszel method was used to calculate the weighted pooled odds ratio under the fixed effects model.

**Results:** A total of 579 results were generated. Duplicates were removed, and 372 records were excluded based on irrelevant abstracts, titles, study design not consistent with the stated outcome, or unavailable full-text. Twelve studies meeting the inclusion criteria were reviewed in full-text; two of these articles were eventually removed due to unconfirmed OSA diagnostic modality, and one was also removed based on a control group inconsistent with the other studies. Therefore, a total of nine studies were included in this meta-analysis for the random pooled effects model (*n* = 19,837). Sample sizes ranged from *n* = 160 patients to *n* = 6841 patients. The risk of AF was found to be higher among OSA versus the control group (odds ratio (OR) = 2.120; confidence interval (CI) = 1.845–2.436; *Z*-score = 10.598; *p* < 0.001). The heterogeneity observed for the pooled analysis was *Q*-value = 22.487; degree of freedom (df; Q) = 8; *p*-value = 0.004; *I*-squared = 64.424; Tau2 = 0.098, suggesting appropriate study selection and moderate heterogeneity. 

**Conclusions:** In conclusion, OSA/SDB is strongly associated with atrial fibrillation as demonstrated by our meta-analysis. Our study confirms and further strengthens the notion that OSA/SDB populations are at high risk for the development of AF and subsequent cardiovascular morbidity and mortality. Prospective studies are needed to ascertain the effect of the treatment of OSA/SDB for the prevention of AF, a growing health burden with serious consequences.

### 2.5. Patiromer for Hyperkalemia Treatment in Resistant Hypertensive Patients on RAASi with Diabetic Kidney Disease

EpsteinMurray^1^MayoMartha^2^GarzaDahlia^2^WilsonDaniel^2^BakrisGeorge^3^1University of Miami, Leonard M. Miller School of Med, Miami, FL 33136, USA2Relypsa, Inc., a Vifor Pharma Group Company, Redwood City, CA 94063, USA3University of Chicago Medicine, Chicago, IL 60637, USA

**Purpose:** Emerging treatments for hyperkalemia (HK) may have a favorable effect on the risk:benefit ratio for patients with hypertension (HTN) and diabetic kidney disease (DKD) treated with renin angiotensin aldosterone inhibitors (RAASi). HK develops in ~7–14% of patients with HTN and DKD, which can limit RAASi dosing and potential cardiorenal benefit. This post hoc analysis from the 52-week AMETHYST-DN study examined the potassium (K^+^)-lowering effect of patiromer, a sodium-free, non-absorbable K^+^-binding polymer approved for the treatment of HK, in resistant HTN (RTN) patients with HK and DKD.

**Methods:** Hypertensive patients with mild (serum K+, >5.0–5.5 mEq/L) and moderate (serum K+, >5.5–6.0 mEq/L) HK and DKD on RAASi were treated with patiromer in an 8-week treatment initiation phase (TIP), followed by a 44-week long-term maintenance phase (LTMP). Safety and efficacy were assessed/analyzed in an RTN cohort (defined at baseline (BL) as systolic blood pressure (SBP) > 140 mmHg on ≥4 classes of antihypertensive medication including a diuretic).

**Results:** In AMETHYST-DN, 79/306 patients (67 ± 7 years, 51% male) had RTN on RAASi—64 with mild HK, mean ± standard error mean (SEM), serum K^+^ = 5.18 (±0.03) mEq/L, and 15 with moderate HK, serum K^+^ = 5.77 ± 0.04 mEq/L. Mean ± standard deviation (SD) heart rate (HR) and SBP/diastolic blood pressure (DBP) were 73 ± 10 bpm and 156 ± 10/83 ± 12 mmHg at BL. At BL, chronic kidney disease (CKD) stage ≥3b was present in 51/79 patients (65%). Significant reductions in serum K^+^ from BL occurred at each time point over the 52 weeks/end of therapy (ET). Investigators could add/modify non-RAASi anti-hypertensive medications, without known effect on serum K^+^, for blood pressure (BP) control. Similar to the BP changes observed in the overall population, at ET the change in mean ± SD SBP/DBP was −18 ± 17/−9.0 ± 13 mmHg in the RTN group. Mean ± SD change in estimated glomerular filtration rate (eGFR) from BL was −2.1 ± 12 mL/min/1.73 m^2^. A total of five out of the 79 subjects withdrew from study due to a patiromer-related adverse event (three due to hypokalemia, and two due to constipation). Doubling of serum Cr occurred in seven patients; one death occurred during the long-term maintenance period(LTMP).

**Conclusions:** Patiromer effectively controlled HK throughout a 52-week period in a high-risk cohort of RTN patients.

### 2.6. The Australian Perspective on Premature Coronary Artery Disease: Prevalence and Risk Factors

GilhotraRitvik^1^GunnarssonRonny^1^,^2^SutcliffeSteven^1^,^2^1James Cook University, Douglas, QLD 4811, Australia2Cairns Hospital, Cairns City, QLD 4870, Australia

**Purpose:** Cardiovascular disease (CVD) is the leading cause of morbidity and mortality in the world as reported by the World Health Organization. CVD is a group of disorders encompassing Coronary Artery Disease (CAD), cerebrovascular disease, and peripheral arterial disease. Shifting our attention to CAD, CAD claimed the lives of over 20,000 Australians in 2012. Not only is the incidence of CAD on the rise, it is also estimated that about 4–10% of individuals with documented CAD are below the age of 45, referring to this phenomenon as Premature Coronary Artery Disease (PCAD). 

Whilst there is a relatively good understanding of CAD in Australia, there is very limited amount of research conducted in the field of PCAD. The reported prevalence of PCAD ranges from 25% to 31% in other countries and as far as we know there is no study in Australia that elaborates on the prevalence of PCAD, let alone the risk factors for PCAD. Furthermore, the true prevalence of PCAD is also believed to be underestimated, as symptomatic CAD at a young age is relatively uncommon. 

The primary outcome of this study was to gain a greater understanding of the prevalence and risk factors for PCAD. The secondary outcomes included the development of a prediction model that could be used to predict the outcome of the coronary angiogram based on an individual’s risk factor profile. Furthermore, this study aimed to determine the factors that correlate to a normal coronary angiogram; in other words, to determine the protective factors against PCAD.

**Methods:** Patients who underwent coronary angiography at the Cairns Hospital between 1 January 2014 and 31 December 2016 were included in the study. The patients had to be equal to or less than the age of 50 years at the time of the procedure.

Patients were excluded from the study if they had multiple coronary angiograms conducted in the time frame (between 1 January 2014 and 31 December 2016). Only the first coronary angiogram conducted on a particular individual in the given time frame was included in the study. 

A National Ethical Application Form (NEAF) was applied to the Far North Queensland Human Research Ethics Committee (HREC). The HREC approval number is HREC/16/QCH/4-1022. Furthermore, a Public Health Act application (PHA) and Site-Specific Application (SSA) was also completed and approved. 

A total of 635 coronary angiograms were conducted at the Cairns Hospital between 1 January 2014 and 31 December 2016 on patients who were ≤50 years of age at the time of the procedure. Eighty-two cases were excluded from the study due to repeated angiograms during this time frame. The final number included in the study was 553 patients. 

Demographic factors included in this study were age, gender, and ethnicity. Clinical risk factors included were history of diabetes mellitus (DM), hypercholesterolemia, smoking, hypertension, family history of CAD, and body mass index (BMI). The biochemical or laboratory risk factors that were included were blood glucose levels, red blood cell count, white blood cell count, neutrophil to lymphocyte ratio, and hemoglobin and creatinine levels.

**Results:** We identified a high prevalence of 64% of PCAD amongst Australian patients undergoing coronary angiography at the Cairns Hospital. Half of the recruited individuals were of Aboriginal and/or Torres Strait Islander background. Furthermore, 57% of the individuals with PCAD in the study were of Aboriginal and/or Torres Strait Islander background. However as per the 2011 census, only 9% of the population in the Cairns region was represented by people with an Aboriginal and/or Torres Strait Islander background. 

We investigated for protective factors against PCAD and identified factors such as increasing age (5-year intervals) (odds ratio (OR) = 0.67 (0.55–0.81; *p* = 0.000026), male gender (OR = 0.40 (0.25–0.65); *p* = 0.00023), Aboriginal and/or Torres Strait Islander background (OR = 0.49 (0.29–0.81); *p* = 0.005), hypercholesterolemia (OR = 0.41 (0.25–0.66); *p* = 0.00027), smoking (OR = 0.45 (0.28–0.73); *p* = 0.001), white cell count (OR = 0.92 (0.86–0.99); *p* = 0.042), and blood glucose levels (OR = 0.91 (0.83–0.99); *p* = 0.032) to be statistically significant after multivariate forward stepwise logistic regression. 

Furthermore, we identified that when comparing the group with pathological coronary angiogram results to the group with normal coronary angiogram results, there was a mean age difference of 2 years, with the pathological group being 2 years older. Similarly, when comparing mean glucose levels within the two groups, the pathological angiogram results group had a mean glucose level of 8.6 mmol/L as compared to a lowly 6.7 mmol/L in the normal results group. The pathological results group also had worse renal function, with a mean creatinine level of 110 umol/L as compared to 85 umol/L in the normal angiogram group. Lastly, the mean neutrophil to lymphocyte ratio, a marker for systemic inflammation, was also higher in the pathological coronary angiogram results group, 8.6 as compared to 6.7 in the normal coronary angiogram results group.

**Conclusions:** We concluded that there is a high prevalence of PCAD within the Cairns region and also amongst the Australian Indigenous patients undergoing coronary angiography at the Cairns Hospital. Other strongly associated risk factors with PCAD include increasing age, male gender, hypercholesterolemia, smoking, white cell count, and blood glucose levels.

### 2.7. Wide Pulse Pressure Associated with Low Bone Mineral Density among Adult USA Population: Analysis of the National Health and Nutritional Examination Survey

McFarlaneIsabel M.AlvarezMilena RodriguezLeonSu ZhazOzeriDavid J.Saladini-AponteCarlaShinTai HoSapersteinYairBhamraManjeetFreemanLatoyaOjikeNwakileDepartment of Medicine, State University of New York, Downstate Medical Center, Brooklyn, NY 11203, USA

**Purpose:** Accumulating evidence indicates an association between osteoporosis and cardiovascular disease (CVD) above and beyond advanced age and estrogen deficiency. Hypertension, a known risk factor for CVD, is also associated with low bone mineral density (BMD) in both men and women, likely related to increased urinary calcium excretion. Brachial-ankle pulse wave velocity is significantly associated with low BMD. In the Rotterdam study and other prospective studies, low BMD was associated with peripheral arterial disease and an age-independent progressive atherosclerosis. In the Multi-Ethnic Study of Atherosclerosis (MESA), low BMD was associated with greater coronary artery calcification as well as increased aortic calcification.

We hypothesize that wide pulse pressure (PP), a strong indicator of CVD risk, will be independently associated with low BMD.

**Methods:** The study used data from two consecutive cycles of NHANES US national health data from 2009–2010 and 2011–2012. NHANES is a complex, multistage, area probability sample representative of the US non-institutionalized civilian population. Point estimates of demographic variables were calculated using the descriptive method. Study participants were divided into four groups based on quartile distribution of PP. Multivariate linear regression analysis was performed to assess the relationship between BMD and PP.

**Results:** A total of 8179 NHANES participants were included in the study. They were 55.2% female. For the entire cohort, the mean age (± standard error mean (SEM)) was 53.3 years ± 0.19, mean body mass index (BMI) (± SEM) was 29.6 kg/m^2^ ± 0.07, and mean PP (mmHg) was 57.2 ± 0.12 and was significantly higher with increased age, among Blacks (58.7 ± 0.18) and Hispanics (57.5 ± 0.19) compared to Whites (53.6 ± 0.16), and for men (57.2 ± 0.16) when compared to women (54.1 ± 0.17), *p* < 0.05. BMD (g/cm^2^) at the lumbar spine was 1.064 ± 0.0026 for men, and 1.00 ± 0.0023 for women. At the femoral neck, BMD was 0.86 ± 0.0026 for men and 0.79 ± 0.0023 for women. After adjusting for age, sex, race, menopause, body mass index, and family history of osteoporosis, PP was associated with femoral neck BMD, β = −0.0005, *p* < 0.05 but was not significantly associated with lumbar spine BMD, β =−0.0002, *p* = 0.07.

**Conclusions:** Our study indicates a negative association between PP and BMD at the femoral neck (high cortical bone content; indicator of senile osteoporosis). This association was not shown at the lumbar spine (high trabecular bone; indicator of postmenopausal osteoporosis). These findings were demonstrated after adjusting for major risk factors for atherosclerosis and osteoporosis, thus indicating an independent association between wide PP and low BMD. Our data show the potential for using PP, a readily available clinical measurement, to identify patients at risk of the low BMD and osteoporosis that is affecting our aging population, leading to increased fractures, disability, and overall mortality.

### 2.8. Multiple-Ascending-Dose Study of IW-1973, a Soluble Guanylate Cyclase Stimulator

HanrahanJohn P.^1^WakefieldJames^1^WilsonPhebe^1^MihovaMarina^1^ChickeringJennifer G.^1^RuffDennis^2^HallMichael^1^MilneG. Todd^1^CurrieMark G.^1^ProfyAlbert T.^1^1Ironwood Pharmaceuticals, Inc., Cambridge, MA 02142, USA2ICON Early Phase Services LLC, San Antonio, TX 78209, USA

**Purpose:** Soluble guanylate cyclase (sGC), an enzyme that catalyzes the formation of cyclic guanosine monophosphate (cGMP) in response to nitric oxide (NO) binding, is a key mediator of local blood flow, inflammation, and fibrosis. IW 1973 is an orally available sGC stimulator that enhances NO sGC-cGMP signaling and reduces blood pressure (BP) in animal models of hypertension, both alone and in combination with other antihypertensive agents.

**Methods:** A phase 1b placebo-controlled, randomized, multiple-ascending-dose study was conducted to assess the safety, tolerability, pharmacokinetics (PK), and pharmacodynamics (BP, heart rate, platelet function, and plasma biomarkers) of IW-1973 in healthy subjects. Four successive cohorts of 11 subjects each (eight active and three placebo) were enrolled. Subjects received a starting dose once daily (QD) for 14 days followed by an up-titrated QD dose for 7 days.

**Results:** IW-1973 doses ranging from 15 to 40 mg were tolerated. There were no serious adverse events (AEs), severe AEs, or discontinuations due to AEs. Among the 32 subjects who received IW-1973, the most common AEs (which occurred mainly at doses ≥30 mg) were headache (15 subjects), dizziness/postural dizziness (six subjects) and orthostatic hypotension/BP decreased (four subjects). AEs tended to resolve with continued dosing. The AE profile of higher doses was not clearly improved by up-titration from a lower starting dose. PK was dose proportional, both for Cmax and area under the curve (AUC), with a Tmax of 2–4 h and an effective half-life of 24–37 h. After 14 days of treatment, least squares mean change from baseline in 24-h ambulatory systolic BP (± standard error (SE)) was 0.85 ± 1.32 (placebo), 7.29 ± 1.62 (15 mg), 3.27 ± 1.61 (20 mg), 6.75 ± 1.62 (30 mg), and 5.23 ± 1.61 mmHg (≤40 mg, ≤0.5 mg/kg). After 21 days, the change from baseline was 4.81 ± 1.19 (placebo), 8.21 ± 1.46 (15 to 30 mg), 6.29 ± 1.45 (20 to 40 mg), 9.05 ± 1.56 (30 to 40 mg), and 6.58 ± 1.45 mmHg (≤40 to ≥40 mg). IW-1973 produced a dose-related increase in plasma cGMP indicating target engagement. There was no clear effect of IW-1973 administration on platelet function as assessed by the PFA-100^®^ system.

**Conclusions:** Further clinical investigation of IW-1973 is ongoing or planned in multiple indications, including hypertension.

## 3. Perspectives

On 4–5 May 2018, CMHC will host its 2nd Annual CMHC West: Advancing Cardiometabolic Health from East to West. Held in Las Vegas. This two-day conference will capture the integrity and high-quality education of the annual Cardiometabolic Health Congress as the nation’s top experts in cardiometabolic health will highlight the latest updates in hypertension, heart failure, diabetes, lifestyle management, and cardiovascular health. The most recent results from top-line clinical trials and new and emerging agents will be brought to you through thought-provoking and innovative education. 

For more information, please visit: https://www.cardiometabolichealth.org/2018/index-west.html.

